# Quantitative Assessment of Stress Through EEG During a Virtual Reality Stress-Relax Session

**DOI:** 10.3389/fncom.2021.684423

**Published:** 2021-07-14

**Authors:** Eduardo Perez-Valero, Miguel A. Vaquero-Blasco, Miguel A. Lopez-Gordo, Christian Morillas

**Affiliations:** ^1^Department of Computer Architecture and Technology, University of Granada, Granada, Spain; ^2^Research Centre for Information and Communications Technologies, University of Granada, Granada, Spain; ^3^Department of Signal Theory, Telematics and Communications, University of Granada, Granada, Spain

**Keywords:** EEG, stress, regression, machine learning, virtual reality

## Abstract

Recent studies have addressed stress level classification *via* electroencephalography (EEG) and machine learning. These works typically use EEG-based features, like power spectral density (PSD), to develop stress classifiers. Nonetheless, these classifiers are usually limited to the discrimination of two (stress and no stress) or three (low, medium, and high) stress levels. In this study we propose an alternative for quantitative stress assessment based on EEG and regression algorithms. To this aim, we conducted a group of 23 participants (mean age 22.65 ± 5.48) over a stress-relax experience while monitoring their EEG. First, we stressed the participants *via* the Montreal imaging stress task (MIST), and then we led them through a 360-degree virtual reality (VR) relaxation experience. Throughout the session, the participants reported their self-perceived stress level (SPSL) *via* surveys. Subsequently, we extracted spectral features from the EEG of the participants and we developed individual models based on regression algorithms to predict their SPSL. We evaluated stress regression performance in terms of the mean squared percentage error (MSPE) and the correlation coefficient (*R*^2^). The results yielded from this evaluation (MSPE = 10.62 ± 2.12, *R*^2^ = 0.92 ± 0.02) suggest that our approach predicted the stress level of the participants with remarkable performance. These results may have a positive impact in diverse areas that could benefit from stress level quantitative prediction. These areas include research fields like neuromarketing, and training of professionals such as surgeons, industrial workers, or firefighters, that often face stressful situations.

## Introduction

Psychological stress is one of the most frequent human affections and has a considerable impact in modern society. According to the 2014 American Psychological Association stress report, about 75% of the population has suffered symptoms linked to stress (American Psychological Association, 2015), and employers spend 300 billion dollars each year in concept of stress related therapies and missed work. Main causes behind stress include job pressure, money, and health condition. In this context, since mental stress is highly correlated with medical issues such as headaches, depression, or insomnia (Marksberry, 2021), stress detection and quantification have recently become active subjects of research.

Recent studies have focused on the early detection of stress in order to prevent more severe health issues. For that purpose, studies generally address stress level detection through multiple biomarkers. For instance, several works have used electroencephalography (EEG)-based features to develop accurate machine learning stress-level classifiers (Jebelli et al., 2018; Asif et al., 2019; Lotfan et al., 2019; Zubair and Yoon, 2020). In these works, researchers typically extract time-frequency features from EEG recordings and split them into training and test sets. Then, they train a classifier using the training set and validate it on the test set. These approaches often achieve remarkable performance, for instance, in Jebelli et al. (2018) and Asif et al. (2019), the authors obtained accuracies of 80.32 and 95.06% for a two-level and three-level stress classifier, respectively. Nevertheless, these works restrict the target space to two or three stress levels, corresponding to low, medium, and high stress. Additionally, performance report is very heterogeneous across studies. Indeed, some of these works only report classification accuracy, overlooking intra-class performance, and they typically develop a single prediction model for all the participants, disregarding participant distinctiveness of brain activity. Alternatively, other works have addressed stress assessment through regression (Park et al., 2018; Ahuja and Banga, 2019; Dimitriev et al., 2019). Conversely to stress classification, these approaches do not aim to detect two or three stress levels, but to examine stress as a continuous variable. However, many of these works only assessed the linear relationship between certain biomarkers and self-perceived stress level (SPSL) (Saeed et al., 2017; Dimitriev et al., 2019), while others presented preliminary quantitative predictions of stress level that yielded middling results (for instance, in Park et al. (2018), the authors obtained a correlation coefficient of 0.64, and in Das et al. (2017), the authors did not provide a scoring metric for their regression predictions, although graphic representation of the predicted and actual stress values showed poor performance).

In this article, our aim is to develop an individualized quantitative stress prediction model using EEG activity and regression algorithms. To achieve this, we developed an individual regression model for each of the participants that predicted their SPSL from their EEG spectral features. To this aim, we conducted a group of participants through a stress-relax procedure. First, we stressed them by means of the Montreal imaging stress task (MIST) (Dedovic et al., 2005), a well-established methodology to elicit psychosocial stress (Dedovic et al., 2005; Freitas et al., 2013; Asif et al., 2019), and then we conducted them through a virtual reality (VR) relaxation experience (Vaquero-Blasco et al., 2021). We recorded the brain activity of the participants across the session with an EEG acquisition system to obtain relevant EEG biomarkers. These biomarkers have been widely used in literature to evaluate stress, and include spectral power in different bands (Schlink et al., 2017; Ahn et al., 2019), asymmetry in the Alpha band (Düsing et al., 2016; Quaedflieg et al., 2016) and Relative Gamma (RG) (Lutz et al., 2004; Steinhubl et al., 2015; Minguillon et al., 2017; Vaquero-Blasco et al., 2020). Additionally, we asked the participants to report their SPSL throughout the session according to an adaptation of the perceived stress scale (PSS) (Cohen et al., 1983). Finally, we used the SPSL surveys and the EEG biomarkers extracted from each participant to train and validate different regression algorithms, namely ridge regression (RR), random forest (RF), multi-layer perceptron (MLP), and support vector regression (SVR), that are usually applied in stress prediction literature (Khosrowabadi et al., 2011; Das et al., 2017; Ahn et al., 2019; Dimitriev et al., 2019).

To sum up, the objective of this work is to provide an individualized quantitative stress prediction model from EEG biomarkers and machine learning regression algorithms. To validate our proposal, we obtained regression performance metrics and we compared our approach with other approximations in literature. Lastly, we believe this work can impact fields like neuromarketing, gaming, and training of certain professionals dealing with stressful situations, as in all of these areas stress assessment may contribute positively.

## Materials and Methods

### Participants

Twenty-three healthy volunteers (14 females, 8 males, and 1 non-binary) were recruited 2 weeks prior to the onset of the study. The age of the participants ranged from 18 to 40 (mean age 22.65 ± 5.48). We only considered participants without health issues and mental disorders. Participants belonged to the community of the University of Granada, and they were not rewarded in any way for their participation. Before the beginning of the study, we briefed the participants about the different phases of the experimental procedure, and we required them not to consume any stimulant nor relaxant the day before the session. Every participant took part in a single 18-min experimental session. The entire data capture spanned for approximately 3 weeks. Since the information derived from the participants was limited to the recording of their brain activity, ethical approval was not required for the present study in compliance with local institutional requirements. Nonetheless, all the participants signed an informed consent prior to the start of the study.

### Experimental Procedure

**FIGURE 1:**
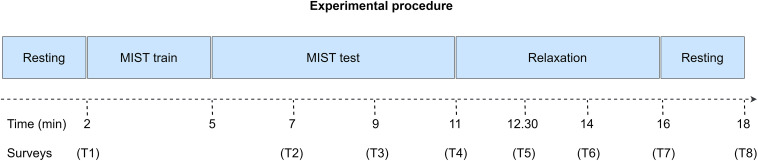
Experimental procedure of the study. Initially, we conducted the participants through a 2-min eyes-closed resting state phase. Secondly, we trained the participants to learn how to use the MIST interface. Then, we conducted them through the MIST, a test designed to induce psychosocial stress *via* arithmetical operations and social pressure. Subsequently, the participants relaxed for 5 min *via* a VR experience. Finally, they completed another 2-min eyes-closed resting state period. The entire procedure spanned for approximately 18 min. Throughout the session, we performed multiple surveys to acquire the SPSL of the participants (T1–T8).

First, we asked the participants to carefully read and sign the informed consent. Then, we equipped them with an EEG cap to acquire their brain activity, and we briefed them about the tasks they had to perform during the experimental session. These tasks are presented in Figure 1.

Along the first phase, we conducted the participants through a 2-min resting state period. During this period, they remained relaxed with their eyes closed. We designed this phase to prompt a baseline relaxation level among the participants. After that, we stressed the participants *via* the MIST, a test designed to elicit psychosocial stress *via* arithmetical operations and social pressure. Subsequently, we led the participants through a 5-min relaxation phase. During this phase, the participants selected their preferred 360-degree virtual experience from a group of four experiences, and underwent it using a VR head mounted display (HMD). Available experiences included a cascade, an aurora borealis, a solitary beach, and a space trip. Throughout this phase, participants remained alone in the room while researchers monitored them from the outside. Finally, during the last phase of the experimental session, participants performed another 2-min resting state period with their eyes closed.

During the entire session, participants were equipped with an EEG acquisition system that recorded their EEG activity. Upon conclusion of the data capture, we extracted EEG features from these recordings to predict the stress level of the participants. Additionally, we conducted several surveys to obtain the SPSL of the participants throughout the experimental session (T1–T8 in Figure 1).

### Experimental Setup

Regarding the MIST, we implemented this test as a graphical interface in MATLAB R2016a (MathWorks). The MIST comprised a series of arithmetical operations including combinations of additions, multiplications, and divisions. To complete this phase, we briefed the participants to use only their dominant hand to work out the operations *via* the touchscreen of the laptop that displayed the MIST interface. We divided the MIST phase into a 3-min training and a 6-min test. Training was designed for the participants to familiarize with the MATLAB interface. During this phase, the participants did not have an imposed time limit to solve each operation, and they were not required to reach a baseline success rate. Conversely, during test, the participants had an imposed time limit to solve each operation that was displayed as a progress bar in the MATLAB interface. Moreover, after each operation, the interface displayed an additional progress bar reporting their success rate. To induce more stress on the participants, researchers required the participants to reach an unfeasible success rate during the test. Additionally, researchers joined the room in three occasions during the test to verbally stress the participant. In relation to the SPSL surveys conducted during this phase (T2–T4), we integrated them in the MIST interface so the participants could answer using the touchscreen of the laptop.

With respect to the relaxation phase, we implemented each virtual experience using a 360-degree video and Unity engine, and then we utilized the Oculus Quest HMD to reproduce the virtual experiences. In the same way we did for the MIST phase, we embedded surveys T5–T7 in the virtual experiences so the participants were able to answer through the HMD controller. Before the start of the relaxation phase, the participants selected one of the four different virtual experiences available, namely a cascade, an aurora borealis, a solitary beach, and a space trip.

In relation to the EEG acquisition, we used the Versatile EEG system, a semi-dry acquisition device designed by Bitbrain (Spain), that works at a sampling rate of 256 Hz. For the electric setup, we considered four electrodes located at positions Fp1, Fp2, F5, and F6 of the 10–20 International System based on previous successful studies about emotions assessment (Brown et al., 2011; Jenke et al., 2014; Vaquero-Blasco et al., 2020). The electrodes were referenced at the left ear lobe and grounded to an extra electrode equidistant from Fpz and Fz. For all the participants, we acquired an independent EEG recording for each phase of the experimental session.

Lastly, regarding the SPSL surveys, we implemented a customized version of the PSS to minimize the time required for the participants to answer. In particular, each survey comprised a single question: “What is your level of stress in a scale from 1 to 5, if 1 is the minimum level and 5 is the maximum level?” We performed the surveys at eight key points of the experimental session to gather a suitable sample of the SPSL of the participants. Concretely, we rendered the surveys as follows: T1, at the end of the initial resting state; T2–T3, 120 and 240 s past the onset of the MIST test; T4, at the end of the MIST test; T5–T6, 90 and 180 s after the start of the relaxation phase; T7, at the end of the relaxation phase; T8, at the end of the second resting state. We did not conduct a survey after the MIST training as we implemented this phase exclusively to brief the participants about the use of the MIST interface.

### Signal Processing

**FIGURE 2:**
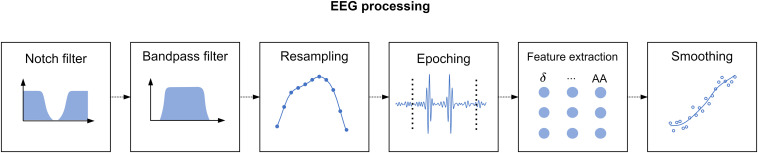
Electroencephalography processing pipeline. First, we applied a notch filter to dismiss the power line artifact. Next, we used a bandpass filter to retain only the desired EEG spectral content. Then, we resampled the filtered signal to match the expected span of the phase under processing. Subsequently, we divided the EEG signals into 2-s epochs. We performed artifact removal, detrending, and standardization at epoch level, and finally we extracted spectral features and smoothed them through a moving-average filter.

Since we acquired a separate EEG recording for every phase of the study, we applied the processing pipeline described in Figure 2 to each of these recordings. Additionally, we withdrew the recordings corresponding to the first resting state, since we intended this phase to induce a baseline relaxation state only, and the MIST training, as we designed this phase only to instruct the participants on the use of the MATLAB interface. In total, the processed recordings spanned for 12 min corresponding to the MIST test (6 min), the relaxation phase (5 min), and the final resting state (we considered only the central minute of this phase).

To process the EEG recordings, first we applied a notch filter with stopband 48–52 Hz to remove electric coupling. Then, we applied a 4th order zero-phase shift Butterworth bandpass filter with bandpass 2–48 Hz to retain the relevant EEG spectral content. Furthermore, it is worth to note that the duration of the EEG recordings of the stages of the study differed by a few seconds across participants. The reason behind is that we marked the onset and the ending of the EEG recordings manually. To overcome this issue, we resampled the filtered signals at a sampling rate such that the length of the resampled signals matched the theoretical length of the stages described in section “Experimental Procedure.” Thereafter, we splitted the filtered signals into 2-s epochs. We tagged the epochs above an amplitude of 75 μV as outliers and zeroed them. We selected this amplitude limit after visual inspection of the signals in accordance to prior studies (Cassani et al., 2017; Jeunet et al., 2020). Then, we detrended and standardized every epoch. To detrend, we removed the best straight-line fit for the epoch from each epoch value. To standardize, we subtracted the epoch mean from each epoch value and divided it by the epoch standard deviation. Subsequently, we carried out a feature extraction phase. In this phase, we estimated the channel-averaged power spectral density (PSD) in Delta (1–4 Hz), Theta (4–8 Hz), Alpha (8–13 Hz), Beta (13–25 Hz), and Gamma (25–45 Hz) bands, the RG, and the Alpha asymmetry (AA). Prior to the estimation of the spectral power, we applied a Tukey window to ease edge effects. Formulae for RG and AA are presented in Eqs 1 and 2. Lastly, we applied a moving average filter with a 30-s window to smooth the extracted features. Figure 2 represents the main stages of the signal processing pipeline described in this paragraph. It is worth to note that, as a result of resampling the processed features had the same length for all the participants. This length was equal to 360 samples, corresponding to the 12-min EEG recording splitted into 2-s epochs (see Eq. 3). Consequently, we obtained a feature matrix with size 360 × 7 (360 samples, 7 features) per participant.

(1) $$R\,G=\frac{P_{G\,a\,m\,m\,a}}{P_{A\,l\,p\,h\,a}+P_{T\,h\,e\,t\,a}}$$

(2) $$A\,A=P_{A\,l\,p\,h\,a\,\left(F\,6\right)}-P_{A\,l\,p\,h\,a\,\left(F\,5\right)}$$

(3) $$360\,e\,p\,o\,c\,h\,s=\frac{60_{\frac{s}{m\,i\,n}}\cdot 12_{m\,i\,n}}{2_{\frac{s}{e\,p\,o\,c\,h}}}$$

RG, P, AA, F6, F5, s, and min stand for RG, Power, AA, F6 electrode positions, F5 electrode position, seconds, and minutes.

**FIGURE 3:**
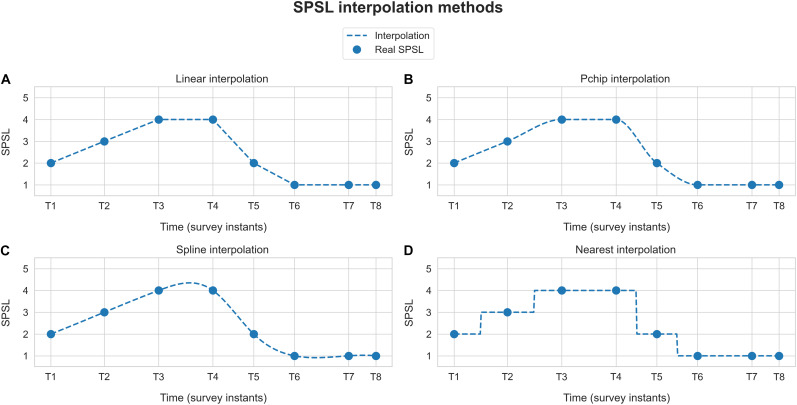
Self-perceived stress level interpolation example. Blue dots represent the responses provided by the participant to the SPSL surveys (T1–T8). Dashed blue line corresponds to the interpolation of the SPSL values. Each graph represents one of the four interpolation schemes applied. **(A)** Linear interpolation, **(B)** pchip interpolation, **(C)** spline interpolation, and **(D)** nearest interpolation.

With regard to stress assessment, as outlined in subsection “Experimental Setup,” we performed eight SPSL surveys at key instants of the experimental session. Since we planned to develop participant-level models, we had only eight target samples per participant at our disposal (T1–T8), in contrast to the 360-sample feature matrix. To overcome this, we interpolated the SPSL surveys of the participants to match the number of samples of the feature matrix. Particularly, we implemented four interpolation methods, namely, linear, pchip (cubic), spline, and nearest interpolation. We applied this procedure based on the hypothesis that emotions cannot drastically change during a small period of time (Taylor, 2006). As an example, Figure 3 illustrates the different SPSL interpolation methods.

### Model Development and Evaluation

**FIGURE 4:**
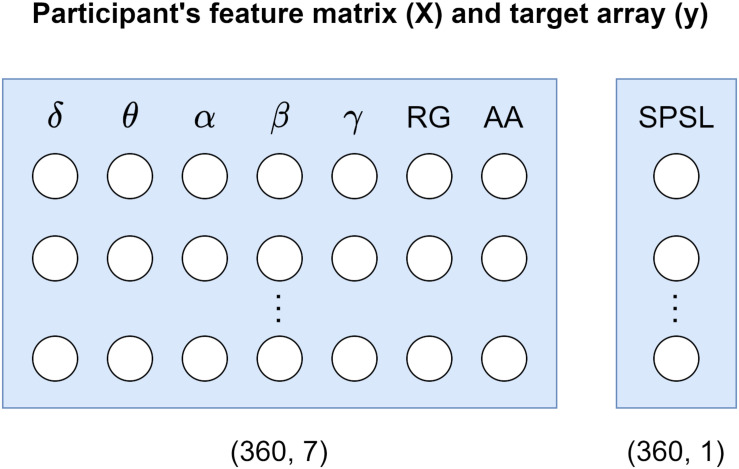
Participant’s data structures utilized for model development. Feature matrix *X* corresponds to the seven spectral features that we estimated from the EEG recordings. Target array *y* refers to the SPSL values that we obtained from the SPSL surveys of the participants through interpolation.

To develop the SPSL regression models we used Scikit-learn toolbox for Python 3. We developed a separate model per each participant. To this end, for each participant, we considered a feature matrix holding 360 samples of the seven spectral features that we estimated, and a target array carrying 360 SPSL samples (eight true SPSL survey answers plus the interpolated data). See Figure 4 for a visual representation of these structures.

**FIGURE 5:**
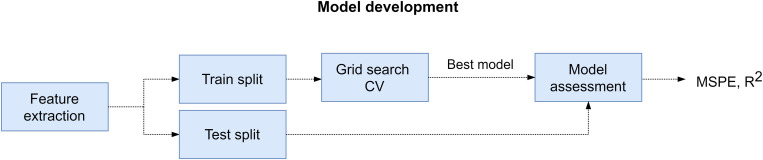
Model development pipeline. First, we extracted feature matrix (*X*) and target array (*y*) from EEG recordings and SPSL surveys, respectively. Later, for every participant, we splitted these data structures into training and test set. Then, we performed grid search cross-validation to find the best set of hyperparameters for the four regressors considered. Subsequently, we refitted the regressor with best hyperparameters on the training set, and we assessed the model on the test set.

The procedure that we followed to develop and evaluate the regression models is described next. First, we extracted the training and test sets from feature matrix (*X*) and target array (*y*) of the participant. For the training set we selected the samples that corresponded to the SPSL points that we interpolated (352 samples). And for the test set we utilized the samples that corresponded to the SPSL answers provided by the participants (T1–T8). Then, we standardized the columns of the feature matrix to normalize the range of the features so they contributed proportionately. Next, we carried out the following procedure for each of the four regressors that we considered in the study, namely RR, RF, MLP, and SVR: we performed grid search cross-validation on the training set to find the best set of hyperparameters for the regressor. During this procedure, we explored multiple combinations of hyperparameters and applied fivefold cross validation to each combination using the training set. After ensuring cross-validation results were not subject to overfitting nor underfitting, we obtained the best set of hyperparameters and we trained the regressor with those hyperparameters and using all the data in the training set. Finally, we evaluated the regressor on the test set in terms of the mean squared percentage error (MSPE) and the Pearson correlation coefficient (*R*^2^). We examined the MSPE as this metric provides a comprehensible measure of the error that does not depend on the amplitude of the target (see Eq. 4). In addition, we examined *R*^2^ to assess the correlation between the real stress values and the predictions. We followed the procedure described in this paragraph to train and evaluate a model for each participant. For a visual representation of this procedure, refer to Figure 5.

(4) $$MSPE=\frac{\frac{1}{n}.\sum_{i=1}^{n}{\left(y_{test_{i}}-y_{pred_{i}}\right)}^{2}}{\frac{1}{n}.\sum_{i=1}^{n}y_{test_{i}}}.100$$

Notice that, since we considered four regressors, we explored different hyperparameters for each of them during grid search cross-validation. The list of hyperparameters that we examined for each regressor is provided in Table 1. For reproducibility, we set random_state variable to zero for all Scikit-learn functions.

**TABLE 1:** Hyperparameters explored using grid search cross-validation for the different regressors examined in the study.

Regressor	Hyperparameters	Values
Ridge regression	Polynomial degree	1, 2, 3
	Regularization penalty	10^–4^, 10^–3^, 10^–2^, 10^–1^, 10, 10^2^
Random forest	Number of estimators	100, 200, 300
	Maximum number of features	5, 6, 7
	Maximum depth	6, 7
Multi-layer perceptron	Number of hidden layers	1, 2
	Number of neurons per hidden layer	3, 4
	Activation function	relu, tanh
	Regularization penalty	10^–3^, 10^–2^, 10^–1^
Support vector regression	Kernel type	Linear, poly, RBF, sigmoid
	Regularization parameter	2^–5^, 2^–3^, 2^–1^, 2, 2^3^
	Epsilon	10^–3^, 10^–2^, 10^–1^

*RBF stands for Radial basis function.*

## Results

Table 2 presents the performance yielded by the different regressors for all the SPSL interpolation methods that we examined in this study.

**TABLE 2:** Performance of the different regressors evaluated in the study.

	Linear		Pchip		Spline		Nearest	
				
	MSPE	*R*^2^	MSPE	*R*^2^	MSPE	*R*^2^	MSPE	*R*^2^
RR	24.96 ± 3.61	0.76 ± 0.03	24.14 ± 3.35	0.76 ± 0.03	24.56 ± 3.41	0.76 ± 0.03	23.65 ± 3.26	0.77 ± 0.03
RF	**10.62 ± 2.12**	**0.92 ± 0.02**	11.11 ± 2.37	0.91 ± 0.02	11.24 ± 2.53	0.91 ± 0.02	11.97 ± 2.91	0.90 ± 0.02
MLP	23.09 ± 3.63	0.77 ± 0.03	22.20 ± 3.37	0.78 ± 0.02	20.14 ± 2.91	0.80 ± 0.02	23.56 ± 3.44	0.73 ± 0.04
SVR	18.97 ± 3.50	0.80 ± 0.04	18.67 ± 3.43	0.80 ± 0.04	17.84 ± 3.66	0.81 ± 0.04	21.46 ± 3.55	0.80 ± 0.03

*Columns display the average mean squared percentage error and Pearson correlation coefficient for each of the four interpolation methods applied. For both metrics we have included also the standard error of the mean (SEM). Rows represent the different regressors that we evaluated. The bold values represent the highest performance yielded in terms of MSPE and R2.*

**FIGURE 6:**
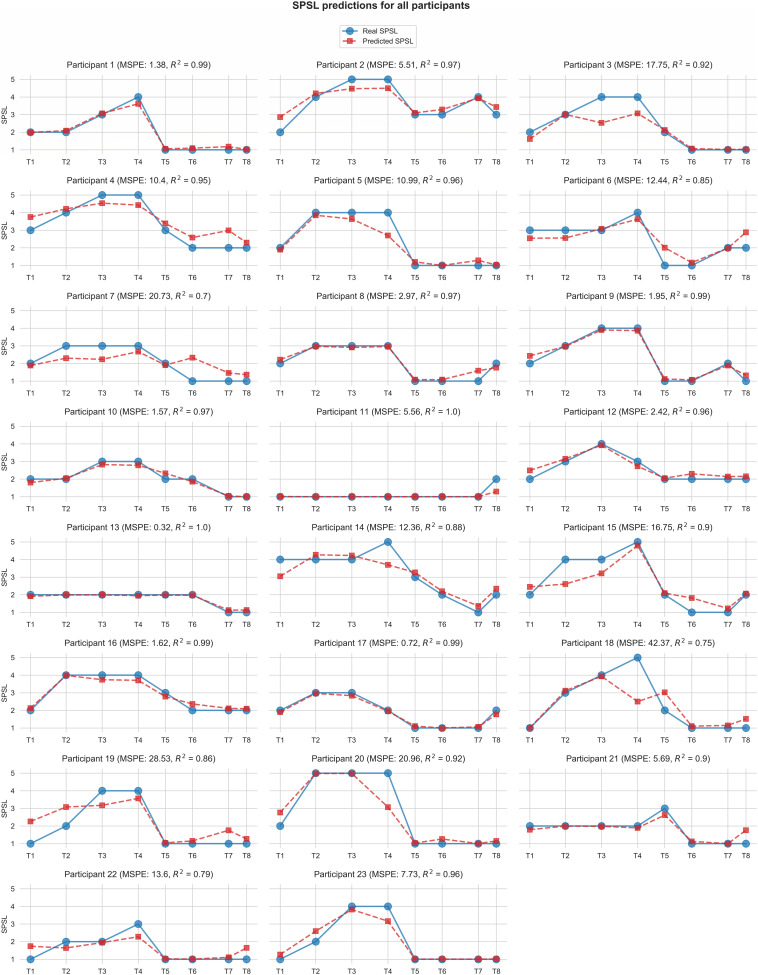
Self-perceived stress level values predicted by the best regression model (random forest with linearly interpolated data) for each participant. Red square markers represent predicted SPSL values in T1–T8. Blue round markers represent SPSL values provided by the participant in T1–T8. MSPE and *R*^2^ for each participant are reported in brackets in the graph titles. For simplicity, we have displayed *Y*-axis only in the first graph of each row, and we have used the same scale for all the participants.

Figure 6 displays the predictions for each of the participants yielded by the best SPSL regression model.

**FIGURE 7:**
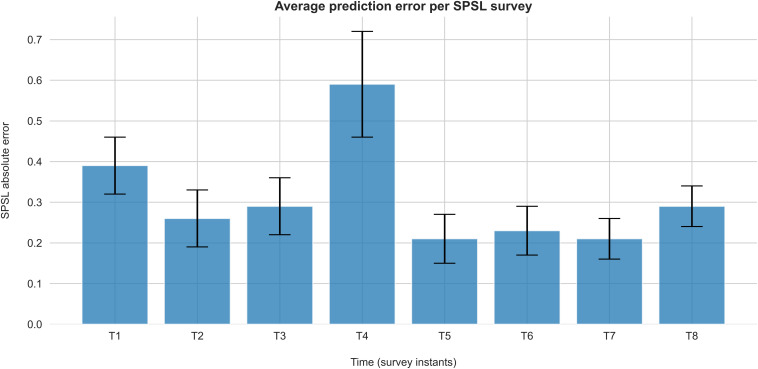
Absolute error of SPSL predictions on each survey for the best regression model (random forest with linearly interpolated data). Each bar represents the average absolute error of the SPSL predictions across the participants. Error bars indicate the SEM.

Figure 7 represents the average absolute error of the predictions at each survey instant for the best regression model.

## Discussion

The goal of this study was to provide a quantitative assessment of the stress level through individualized regression algorithms. To this end, we gathered the SPSL of a group of participants throughout a stress-relax procedure, and we evaluated different regressors based on EEG spectral features. Instead of fitting a single regressor to the data from all the participants, we fitted a regressor per participant. The main reason behind this argument is the well-known inter and intra-participant particularities observed in EEG due to non-stationary brain activity. The results that we obtained indicate that regression models can quantitatively predict stress level with noteworthy performance. On the one side, the high correlation coefficient (0.92) obtained by the best-performing regressor (random forest) evidences the ability of this model to track the progression of the SPSL throughout a stress-relax session. Moreover, the low MSPE (10.62) yielded by this regressor demonstrates its capability to produce quantitative predictions of the SPSL with remarkable performance, what fulfills our expectations in regards to stress assessment.

According to Table 2, random forest regression from linearly interpolated SPSL data yielded the best performance in terms of MSPE (10.62) and *R*^2^ (0.92). Figure 6 evidences that, in general, random forest regression accurately predicted the SPSL values for all the participants. Indeed, this regression model predicted very different SPSL patterns with remarkable performance, from steady progressions such as participant 13, to more intricate progressions such as participants 9 and 5. The notable performance of this model is further evidenced in Figure 7. This figure indicates that the mean absolute error among the different surveys was lower than 0.6 for all surveys. It is also notable that the higher error occurred at T4, that corresponds to the last survey of the MIST test phase. This may be explained by the fact that participants often reached their peak SPSL at the end of the MIST, while for the rest of the surveys the SPSL followed a steadier progression.

The results that we obtained in this study support other works that assessed the feasibility of predicting stress from EEG features. Most part of these studies addressed stress prediction as a classification task. Nevertheless, this approach is limited to the detection of two or three classes (typically low stress, medium stress, and high stress) (Wijsman et al., 2011; Schlink et al., 2017; Attallah, 2020). Furthermore, performance is usually non-uniformly reported among studies, with some authors often reporting only classification accuracy, hence disregarding class balance (Jebelli et al., 2018; Lotfan et al., 2019). As a result, the outcomes of these studies are hardly comparable. Alternatively, we approached stress prediction as a regression task to broad the target space to continuous values of stress, and provide a more interpretable performance metric (prediction percentage error). In this context, other authors have evaluated stress level regression from features such as EEG spectral power or heart rate variability (HRV). For instance, in Park et al. (2018), authors assessed self-perceived stress predictions through linear regression of an HRV spectral feature. However, they did not evaluate the linearity between the predictor and the target, and restricted their analysis to a scatter plot. As a result, the model yielded poor performance for some of the participants. Furthermore, they computed accuracy after scaling the prediction error by the maximum value of the PSS scale (40), although some of the participants did not reach a level of stress even close to this value, what led to unrealistic high accuracy for some of them. In contrast, we normalized the error by the average value of the SPSL, what results in a more realistic performance report (see Eq. 4). Other authors have assessed stress level regression from EEG features, nevertheless, they often report their results in terms of *R*^2^, hence disregarding the quantitative error between the predictions and the real stress levels. For instance, in Saeed et al. (2017) and Das et al. (2017), the authors used a regression model to predict stress level, however, they only reported performance in terms of *R*^2^ (0.13 for the first study, and close to 0.80 for the second). Conversely, we supported the correlation score provided by *R*^2^ with an error score like the MSPE (although other metrics like the mean squared error of the mean absolute error may be considered). Additionally, in Das et al. (2017), although the authors applied a non-linear regression model (random forest), they did not report a procedure for estimating the hyperparameters of the regressor. Such a procedure is crucial to optimize the prediction capabilities of the model. We applied grid search cross-validation to this end, but other methods like random search also achieve remarkable performance at hyperparameter optimization (Bergstra and Bengio, 2012).

It is worth noting that we designed the SPSL surveys to minimize the interferences during the experimental session. However, a longer session that included a higher number of surveys may improve stress predictions, as the dataset would rely on interpolated data to a lower extent compared to the present study. In addition, although we believe our results are good enough to support our conclusions, increasing the number of participants would likely reduce the SEM of the predictions.

Finally, multiple fields could take advantage of the stress regression approach that we propose in this study, as it only requires an 18-min calibration to gather data to train the participant-individualized model and few frontal electrodes. For instance, emotion prediction based on psychophysiological measures is a conventional target in neuromarketing (Kumar, 2015; Garczarek-Ba̧k et al., 2021). In this scenario, an unobtrusive approach like the one presented in this article may provide insights about the decisions made by consumers and their reactions to certain products. Alternatively, for professionals working under stressful conditions such as firefighters, industrial workers, or surgeons, stress quantitative assessment may be useful during training (Chrouser et al., 2018; Oskooei et al., 2021). Thereby, individual stress models of the trainees could provide the instructors valuable stress biofeedback that may be used to support education techniques. Moreover, our approach could be also accommodated in gaming, where ubiquitous biofeedback is gaining popularity recently (Osman et al., 2016; Kerous et al., 2018). In that direction, quantitative predictions of the stress felt by the players may be utilized to anticipate their actions or modulate game difficulty. Lastly, further studies must evaluate the use of different electric setups and alternative EEG features, such as coherence among different brain areas and synchronization, as these aspects may enhance the prediction capabilities of the stress regression models.

## Data Availability Statement

The raw data supporting the conclusions of this article will be made available by the authors, without undue reservation.

## Ethics Statement

Ethical review and approval was not required for the study on human participants in accordance with the local legislation and institutional requirements. The patients/participants provided their written informed consent to participate in this study.

## Author Contributions

EP-V and ML-G: conceptualization. EP-V, MV-B, and ML-G: methodology. EP-V and MV-B: software, formal analysis, investigation, and writing—original draft preparation. EP-V, MV-B, ML-G, and CM: validation and writing—review and editing. CM and ML-G: resources, supervision, and project administration. EP-V: data curation and visualization. All authors have read and agreed to the published version of the manuscript.

## Conflict of Interest

The authors declare that the research was conducted in the absence of any commercial or financial relationships that could be construed as a potential conflict of interest.
